# Impaired burrowing is the most prominent behavioral deficit of aging htau mice

**DOI:** 10.1016/j.neuroscience.2016.05.004

**Published:** 2016-08-04

**Authors:** Philippine Camilla Geiszler, Matthew Richard Barron, Marie-Christine Pardon

**Affiliations:** Neuroscience Group, School of Life Sciences, University of Nottingham, Queens Medical Centre, Nottingham NG7 2UH, United Kingdom

**Keywords:** 4R, 4-repeat, AD, Alzheimer’s disease, ANOVA, analysis of variance, MAP1A, microtubule-associated protein 1A, mtau^−/−^, murine tau knockout, NFTs, neurofibrillary tangles, RIPA, radioimmunoassay precipitation, htau, tau knock-out, cognition, behavior, tauopathy, Alzheimer’s disease

## Abstract

htau mice are deficient of murine tau but express all six human tau isoforms, leading to gradual tau misprocessing and aggregation in brain areas relevant to Alzheimer’s disease. While histopathological changes in htau mice have been researched in the past, we focused here on functional consequences of human tau accumulation.

htau mice and their background controls – murine tau knock-out (mtau^−/−^) and C57Bl/6J mice – underwent a comprehensive trial battery to investigate species-specific behavior, locomotor activity, emotional responses, exploratory traits, spatial and recognition memory as well as acquisition, retention and extinction of contextual fear at two, four, six, nine and twelve months of age.

In htau mice, tau pathology was already present at two months of age, whereas deficits in food burrowing and spatial working memory were first noted at four months of age. At later stages the presence of human tau on a mtau^−/−^ background appeared to guard cognitive performance; as mtau^−/−^ but not htau mice differed from C57Bl/6J mice in the food burrowing, spontaneous alternation and object discrimination tasks. Aging mtau^−/−^ mice also exhibited increased body mass and locomotor activity.

These data highlight that reduced food-burrowing performance was the most robust aspect of the htau phenotype with aging. htau and mtau^−/−^ deficits in food burrowing pointed at the necessity of intact tau systems for daily life activities. While some htau and mtau^−/−^ deficits overlap, age differences between the two genotypes may reflect distinct functional effects and compared to C57Bl/6J mice, the htau phenotype appeared stronger than the mtau^−/−^ phenotype at young ages but milder with aging.

## Highlights

• htau mice exhibit robust deficits in food burrowing.
• Behavioral differences between htau and mtau^−/−^ are age-dependent.
• Before 6 months of age, the htau phenotype is stronger than the mtau^−/−^ phenotype.
• With aging, the htau phenotype is milder than the mtau^−/−^ phenotype.

## Introduction

Tau is a microtubule-associated protein (MAP) which regulates microtubule dynamics and transport of mitochondria in neuronal axons (Medina and Avila, 2014). Tau hyperphosphorylation causes its pathological aggregation and loss of its physiological function (Johnson and Stoothoff, 2004); resulting in the formation of neurofibrillary tangles (NFTs), a hallmark of a number of neurodegenerative diseases termed as tauopathies, including Alzheimer’s disease (AD) (Lee et al., 2001). NFTs are reported to be correlated to AD severity (Monsell et al., 2013).

In human adults, tau is present in six isoforms characterized by the presence of with one or two N-terminal inserts and three or four C-terminal repeats which are referred to as 3-repeat (3R) and 4-repeat (4R) tau (Buee et al., 2000). In contrast, adult murine tau only consists of the 4R isoforms which do not hyperphosphorylate (Goedert and Jakes, 1990, Andorfer et al., 2003). Genetically altered human tau (htau) mice are void of murine tau but express all six, non-mutated, human tau isoforms instead (Andorfer et al., 2003). This is of specific relevance to AD which is not associated with mutated forms of tau (Andorfer et al., 2003). In accordance with the histopathological progression of AD, htau mice display an age-related increase of hyperphosphorylated tau in cortical and hippocampal areas; somatic redistribution of tau, indicated by the presence of insoluble tau in dendrites, as early as at two months of age and accumulation of phosphorylated tau in the cell body at three months of age; tau aggregates are observed by nine months of age, resembling later stage NFTs in humans (Andorfer et al., 2003). In addition, htau mice display an increasing inflammatory phenotype from three months of age onward (Bhaskar et al., 2010, Garwood et al., 2010) as well as decreasing cortical thickness from ten-months-of-age onward, and neuronal cell loss at seventeen months of age (Andorfer et al., 2005).

Although these histopathological observations suggest that the htau mouse line recapitulates the tau pathology seen in AD, very few studies have examined the behavioral and cognitive phenotype of htau mice; with Polydoro et al.’s report (2009) being the most extensive. As most prominent cognitive features, spatial learning and recognition memory impairments have been reported in six- and twelve-month-old htau mice compared to non-littermates wild-type C57Bl/6J mice (Polydoro et al., 2009, Phillips et al., 2011).

The aim of the present study was to characterize the progressive behavioral phenotype of htau mice using a trial battery, addressing species-specific behavior, locomotor activity, anxiety-related behavior, learning and memory performance. Two-, four-, six-, nine- and twelve-month-old mice were studied corresponding to advancing stages of tau pathology in htau mice (Andorfer et al., 2003). Unlike previous studies, htau mice were compared to both their murine tau knockout (mtau^−/−^) littermates and to C57Bl/6J mice, given that htau mice were bred on a C57Bl/6J background and removal of murine tau is necessary for tau pathology to occur (Andorfer et al., 2003). While initially thought asymptomatic, mtau^−/−^ mice were recently found to exhibit altered motor functions compared to C57Bl/6J mice (reviewed by Ke et al. (2012)). Since htau mice are bred on a mtau^−/−^ background through the crossing of mtau^−/−^ and htau mice, wild-type littermates are not produced and therefore non-littermates C57BL6J are used as control in behavioral studies (e.g. (Polydoro et al., 2009, Phillips et al., 2011)). Thus, the present study was designed to assess both the gain-of-pathological-tau function and the loss-of-physiological-tau function in htau mice (Johnson and Stoothoff, 2004) and, therefore, to also address the question of whether the behavioral phenotype of htau mice resembles that of mtau^−/−^ mice. Our main finding was that impaired food burrowing is the most robust behavioral deficit of aging htau mice, but subtle phenotypic differences between htau and mtau^−/−^ mice suggest that the two mutations have distinct functional consequences.

## Experimental procedures

### Animals

htau mice and their mtau^−/−^ littermates (STOCK *Mapt^tm1(EGFP)Klt^* Tg(MAPT)8cPdav/J) were purchased from the Jackson Laboratories (Bar Harbor, ME, USA) and bred in the transgenic facility at the University of Nottingham Biomedical Service Unit to produce experimental animals. These mice were backcrossed to C57Bl/6J for ten generations. Like in previous reports (Polydoro et al., 2009), age-matched C57Bl/6J mice served as control animals. Since htau mice were bred on a mtau^−/−^ background, precluding the generation of C57Bl/6J mice as littermates, C57Bl/6J mice were purchased a month prior to the experiments (Charles River, UK). The validity of their use was confirmed by their food-burrowing performance which did not differ from that of C57Bl/6J mice bred in-house (unpublished results). In total, 203 male mice (75 C57Bl/6J; 63 mtau^−/−^ and 65 htau mice) were used and maintained under standard husbandry conditions on a 12/12-h light cycle, with lights on at 07:00 h and room temperature, relative humidity and air exchange automatically controlled. Animals were singly housed in individually ventilated cages with *ad libitum* access to food and water, and provided with nesting material and a play tube.

All procedures were authorized and approved by the University of Nottingham ethics committee and were performed according to the UK Animals (Scientific Procedures) Act 1986, under Home Office project license 40/3601.

### Experimental design

**Fig. 1:**
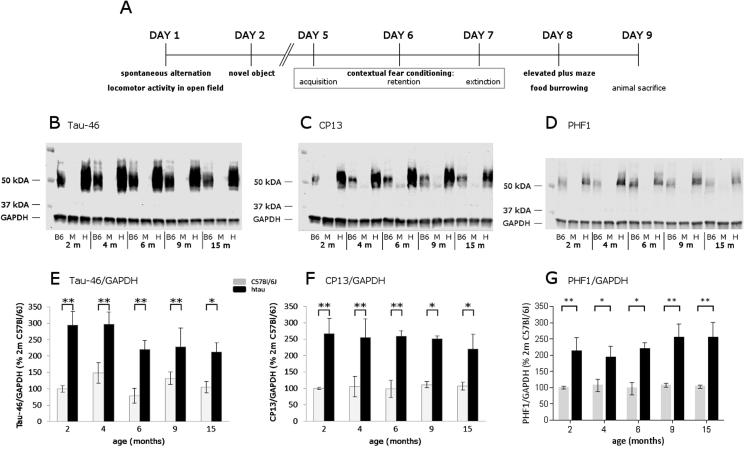
(A) Illustrates the timeline of behavioral testing. Western blots (B–D) of cortex homogenates of two-, four-, six-, nine- and fifteen-month-old C57Bl/6J, mtau^−/−^ and htau mice: showing the absence of total tau (Tau-46) and phosphorylated tau (CP13, PHF1) in mtau^−/−^ mice. Concentrations of total (E) and phosphorylated (F, G) tau were normalized to levels of two-month-old C57Bl/6J mice. Compared to C57Bl/6J mice, htau mice displayed significantly higher levels of phosphorylated and total at all age groups measured. KEY: B6 C57Bl/6J, M mtau^−/−^, H htau, m months-of-age; *n* = 4 per age and genotype.

Independent groups of mice were tested at two-, four-, six-, nine- or twelve months of age in a battery of behavioral and cognitive tests over eight days (Fig.1A). The number of mice used in each experimental condition (*n* = 13) was determined by power calculations based on unpublished food-burrowing data and a published report (Deacon et al., 2002). The actual numbers (*n* = 8–19; Table 1) varied due to the occurrence of dermatitis in some older mtau^−/−^ mice leading to their exclusion from the study and their replacement while controlling for genotype and age. The behavioral and cognitive tasks examined species-specific behavior (food burrowing), locomotor activity and anxiety-related behavior (open-field and elevated plus maze) as well as learning and memory function (spontaneous alternation, novel object location, novel object discrimination, contextual fear conditioning). The order of the tasks was the same for each mouse. Test order was not found to affect performance for the behaviors assessed in our study, and tests were run from the least to most invasive as recommended (McIlwain et al., 2001), albeit food-burrowing behavior was tested last. The InVivoStat software v. 3.2 (Clark et al., 2012) was used for all data analyses except for one-sample *t*-tests which were calculated using SPSS (IBM SPSS Statistics, v. 21).

### Western immunoblotting

To verify the tau pathology, western blotting of total (Tau46), early (CP13) and late (PHF1) pathological stage tau phosphorylation was carried out on cortical tissue of four mice per age and genotype. Samples obtained from twelve-month-old mice were kept to fifteen months of age but not tested behaviorally at this age. All mice’s cortices were homogenized in 5× cold radioimmunoassay precipitation (RIPA) buffer plus inhibitors and centrifuged at 20,000 G at 4 °C for 20 min. The RIPA buffer contained: 50 mM Tris, pH 7,4, 0.1% triton X, 0,25% Na-Deoxycholate, 1 mM EDTA, 1 mM Na_3_VO_4_, 1 mM NaF, 80 mM β glycerophosphate (all: Sigma Aldrich), 150 mM NaCl (Fischer Chemical) and complete protease inhibitor (EDTA free, Roche).

Total protein levels were determined using a Bicinchoninic Acid (BCA) Protein Assay kit (Novagen). Samples were diluted 1:1 in 2× Laemmli buffer (Sigma Aldrich) and heated at 100 °C for 5 min. 15 μg of protein were loaded onto 7.5% Criterion TGX (BioRad Laboratories) gels and transferred to nitrocellulose membranes (Amersham Hybond-ECL, GE Healthcare). Membranes were blocked in 5% milk in TBST (0.1% tween 20) and probed with anti-rabbit GAPDH (1:40,000, G9545; Sigma Aldrich), anti-mouse Tau-46 (1:500, T9450; Sigma Aldrich), anti-mouse CP13 (1:500, Ps202) and anti-mouse PHF1 (1:500, Ps396/404, the latter two were generously gifted by Prof Peter Davies, (NY, USA). IRDye 680RD Goat anti-Rabbit (1:15,000, 926-68071; LI-COR) and IRDye 800CW Goat anti-Mouse (1:15,000, 926-32210; LI-COR) served as secondary antibodies.

Blots were scanned using a LI-COR Odyssey infrared scanner and analyzed using the Odyssey infrared imaging software system (V3). The measured intensities were normalized to GAPDH values, expressed as percent changes from values obtained from two-month-old C57Bl/6J mice and statistically compared by a two-way analysis of variance (ANOVA), with age and genotype as between-subject factors, followed by *post-hoc* planned comparisons. The ratio of CP13 and PHF1 to total tau levels was calculated and likewise analyzed.

### Body mass

All mice were weighed three times during the test week: prior to behavioral testing on Day 1, one day after acquisition of contextual fear conditioning (Day 6) and after the food-burrowing task (Day 8). There was no significant change in body weight within the week, indicating that the trial battery had no gross impact on physical health and a minimal effect on stress. Body mass data collected on Day 8 were used for the statistics. Data were analyzed by a two-way ANOVA, with age and genotype as between-subject factors, followed by *post-hoc* planned comparisons.

### Behavioral testing

#### Spontaneous alternation task (Day 1)

As described previously (Typlt et al., 2013), mice were allowed to freely move in a Y-shaped maze made of transparent plexiglass (44 cm long × 7 cm wide × 25 cm high; three identical arms 120° apart) for five minutes. The number of arms visited was used as a measure of locomotor activity. The number of alternations was manually scored and expressed as a percentage. The latter served as a measure of spatial working memory (Hughes, 2004). Only entries into an arm that differed from the previous two were accepted as successful alternation. The number of arms entered was statistically analyzed by a two-way analysis of covariance (ANCOVA) with genotype and age as between-subject factors and distance moved in the open field trial as covariate. The alternation rate was subjected to a two-way ANCOVA using age and genotype as between-subject factors and the number of arm entries as covariate. *Post-hoc* planned comparisons were used when appropriate. Since each mouse had the choice of entering two different arms once it had left an arm, an alternation rate of 50% was defined as random chance, while an alternation rate of above random chance (50%) indicated the use of a spatial working memory strategy (Gerlai, 2001). This was assessed using one-sample *t*-tests (compared to chance level).

#### Open-field, novel object location and discrimination tests (Days 1 and 2)

Mice were habituated to the open field arena (dark-gray plexiglass floor, surrounded by transparent plexiglass walls, 30 cm long × 35 cm wide × 30 cm high) wherein novel object tests would also be carried out later. During this 30-min-long trial the mice were allowed to move freely. The total distance they covered in the whole arena and in the center was automatically tracked using the Ethovision software (v. XT7, Noldus, Wageningen, Netherlands). The latter parameter was expressed as percentage activity in the center, and both were understood as measures of locomotor activity and emotionality, respectively (Karl et al., 2003).

On the following day, mice were subjected to the novel object location and discrimination tests. Two wooden objects (either octagonal or triangular prisms, in distinct blue-white and red-white patterned color, respectively) were placed into the same open field arena. Their location in the three consecutive six-minute-long trials is depicted in Rattray et al. (2009). In the acquisition trial, each mouse was allowed to explore two identical objects that were located in adjacent corners of the arena. After a 20-min-long interval each mouse was placed into the same arena for the location trial, with one of the two objects moved to another corner of the arena so that the two objects were diametrically opposed. After a three-hour-long inter-trial interval each mouse was allowed to explore the objects for a third time (discrimination trial), albeit one object had been exchanged, *i.e.* the triangular for the octagonal prism or *vice versa*. Care was taken to avoid side preference biases. The time the mice spent exploring the objects was scored manually. Whether they showed a preference for the novel location (trial 2) or object shape (trial 3) was determined by the preference index defined as the percentage time spent exploring the object at the novel location, or the novel shape.

Open field data, total object exploration times and preference indices were analyzed using a two-way ANCOVA, with age and genotype as between-subject factors and the following covariates: body mass (open field data), distance moved in open field (novel object exploration times) and novel object exploration times of each trial (respective preference indices). *Post-hoc* planned comparisons were carried out where appropriate. The preference indices determined from the object tests were also compared to chance levels (50%) using one-sample *t*-tests.

#### Contextual fear conditioning (Day 5, 6 and 7)

For the contextual fear-conditioning task mice were placed in a dedicated arena (25 cm long × 22 cm wide × 38 cm high; three stainless steel, one transparent Plexiglas wall; stainless steel rod floor with rods spaced 1.0 cm apart) in three consecutive trials, 24 h apart. During the acquisition trial, mice were allowed to explore the arena for one minute before they received the first of five foot shocks *via* the steel rod floor (0.4 mA, 1 s, 1 per minute; Camden Instruments, Loughborough, UK). In the following two-minute-long retention and extinction trials no foot shock was applied. The time the mice spent immobile was automatically tracked by Ethovision, whereby a change of mouse “area” of 0.75% as viewed from above was set as threshold of immobility. Increasing immobility time during the repeated shock administration in the first trial reflected successful acquisition of contextual fear. The time spent immobile during the retention and extinction trials indicated contextual fear memory (Bonardi et al., 2011). The extinction index was calculated by subtracting immobility levels measured during trial 3 from those recorded in trial 2.

Immobility times in the acquisition trial were analyzed using a three-way repeated measures mixed model with time as within-subject factor, genotype and age as between-subject factors and distance moved in the open field as covariate. The effects of genotype and age on retention and extinction of contextual memory were determined by a two-way ANCOVA with genotype and age as between-subject factors and immobility during the acquisition trial as covariate; followed, where appropriate, by planned comparisons. Successful extinction of contextual memory was reflected by a significantly negative extinction index value which was assessed by one-sample *t*-tests (compared to 0).

#### Elevated plus maze test (Day 7)

The arms of the elevated plus maze were 5.2 cm wide, 35.2 cm long and the walls along the closed arms 21 cm high and made of opaque white Plexiglas. Mice were allowed to freely explore all arms for 5 min. The number of mice that fell off the maze within the five-minute-long trial was unusually high, which may be due to the relatively large size of the mice, but the width of the open arms conformed with standard instrument set-ups (Carola et al., 2002, Walf and Frye, 2007). All mice that fell off the maze were excluded from the analysis since performance on the elevated plus maze is not repeatable within a short period of time (Walf and Frye, 2007). Ethovision was used to track the time the mice spent in the center of the plus maze which served as index for risk assessment, and the time the mice spent on the maze’s arms to allow the calculation of the percentage time spent in the open arms (*vs.* closed arms) as an indication of anxiety-related behavior (Dawson and Tricklebank, 1995).

Data were analyzed using a two-way ANCOVA, with age and genotype as between-subject factors and body mass or distance moved in the open field arena as covariate (for time spent on open arms and in the center, respectively). Data from the relative time spent on the open arms were log-transformed to normalize the distribution and planned comparisons followed ANCOVA where appropriate.

#### Food-burrowing task (Day 8)

For the species-specific food-burrowing task (Deacon et al., 2002), a glass jar filled with 30 g of ∼1 cm^3^ pelleted food was placed in the mice’s home cage at 05.00 pm and removed the following morning at 09.00 am. The mass of food displaced overnight was weighed.

The impact of genotype and age on food displacement was analyzed by a two-way ANCOVA using age and genotype as between-subject factors and the frequency of testing as covariate to account for potential learning effects in mice that were repeatedly tested. The ANCOVA was followed by *post-hoc* planned comparisons.

## Results

Main statistical effects are reported in Table 2 and summarized in Table 3. Data are presented as mean ± standard error of mean.

### Western immunoblotting

As expected mtau^−/−^ were devoid of total (Tau-46; Fig. 1B) and phosphorylated tau (CP13 and PHF1; Fig. 1C and D). Semiquantitative analysis of the western blots showed that htau mice expressed more total (Fig. 1E) and phosphorylated (Fig. 1F and G) tau than C57Bl/6J mice irrespective of their age (*F*(1,27) = 62.53, *p* < 0.0001 for Tau-46, *F*(1,27) = 42.08, *p* < 0.0001 for CP13 and *F*(1,30) = 46.52, *p* < 0.0001 for PHF1), but the CP13/Tau-46 and PHF1/Tau-46 ratios did not differ between htau and C57Bl/6J mice (*F*(1,27) = 0, *p* = 0.9943 for CP13/Tau-46 and *F*(1,29) = 0.1, *p* = 0.7578 for PHF-1/Tau-46; data not shown); as was the case for the case for PHF1/Tau-46 ratio (*F*(1,29) = 0.001, *p* = 0.9566, data not shown). No significant age or age × genotype interaction effects were found for either Tau-46 and CP13 levels or the CP13/Tau-46 and PHF1/ Tau-46 ratios, data not shown).

This is in apparent contrast with illustrative examples of immunoblots from htau brains which suggest that CP13 increases with age while total tau and PHF1 remains stable, although this was not supported by semiquantitative analyses (Andorfer et al., 2003). It should however be noted that htau mice in Andorfer et al. (2003) were maintained on a mixed genetic background, while the mice used here were fully backcrossed on a C57Bl/6J background. Differences in the genetic background can contribute significantly to phenotype variability (Doetschman, 2009) and affect the severity of the pathology in genetic mouse models of AD (Hall and Roberson, 2012). Thus, the C57BL/6J background may attenuate the phenotype of htau mice.

### Body mass

**Fig. 2:**
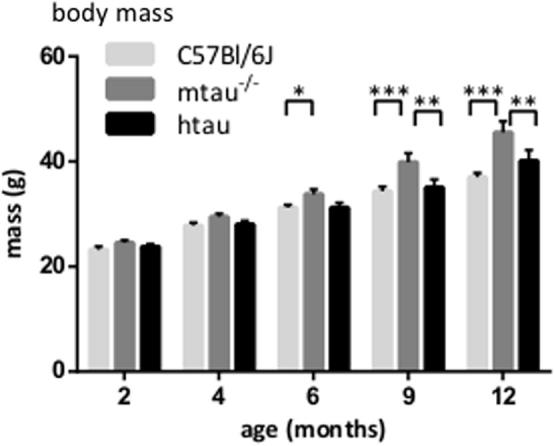
Increase in body mass recorded with aging, whereby mtau^−/−^ mice were significantly heavier than C57Bl/6J and htau mice from six and nine months of age onward, respectively. KEY: ^*^p < 0.05, ^**^p < 0.01, ^***^p < 0.0001 (planned comparisons); *n* = 8–18 per age and genotype.

Body mass increased significantly with genotype in an age-dependent manner (*F*(8,188) = 2.27, *p* = 0.0245). mtau^−/−^ mice weighed significantly more than C57Bl/6J at 6 months of age (*p* < 0.05 in both cases) and more than both C57Bl/6J and htau mice at nine and twelve months of age (*p* < 0.0001 *vs.* C57Bl/6J and *p* < 0.01 *vs.* htau at both age-groups) (Fig. 2).

### Behavioral testing

#### Spontaneous alternation

**Fig. 3:**
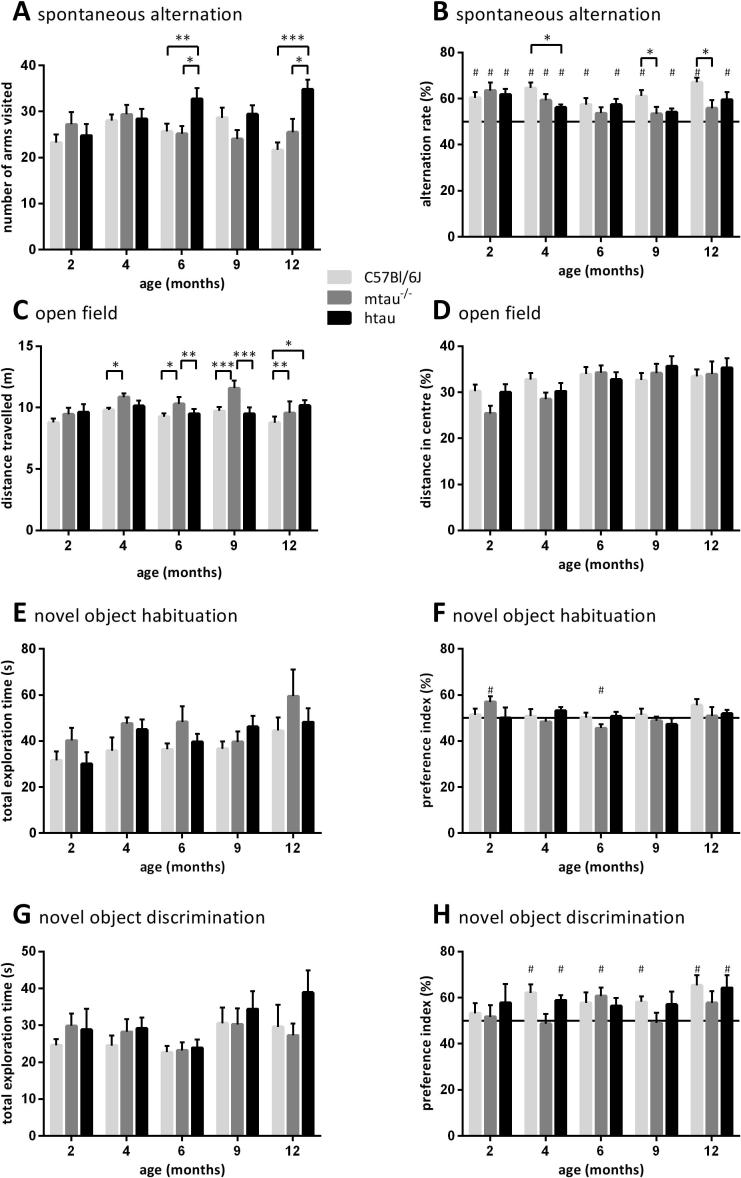
Behavioral performance in the (A, B) spontaneous alternation and (C, D) open field as well as novel object habituation and discrimination (E–H) tasks. (A) During the five-minute-long trial in the Y-maze, htau mice visited significantly more arms than both C57Bl/6J and mtau^−/−^ mice at six and twelve months of age. (B) Spatial working memory performance of htau mice was significantly lower than the one of C57Bl/6J mice at four months of age, while mtau^−/−^ mice were impaired at nine and twelve months of age; as determined by the alternation rate (whereby the 50% line indicates chance level). (C) At nine months of age, mtau^−/−^ mice were significantly more active that both C57Bl/6J and htau mice in a 30-min-long open-field session. (D) There were no differences between experimental groups for the relative distance moved in the center relative to the total area, suggesting that anxiety-related behavior was unaltered by age or genotype. (E, G) The genotypes did not consistently differ from each other in exploring two objects in the (E) habituation or (G) discrimination trial. (F) Object preference during the acquisition of the object discrimination task. (H) mtau^−/−^ mice did not choose to explore a novel object over a familiar object at four, nine, and twelve months of age. KEY: ^*^p < 0.05, ^∗∗^*p* < 0.01, ^***^p < 0.0001 (planned comparisons); ^#^*p* < 0.05 difference from 50% (one-sample *t*-test); *n* = 8–19 per age and genotype.

The number of arms visited differed significantly between genotypes as function of age (*F*(8,177) = 2.36, *p* = 0.0193; Fig.3A). htau mice visited more arms than both C57Bl/6J and mtau^−/−^ mice at six- (*p* < 0.01 and *p* < 0.05, respectively) and twelve months of age (*p* < 0.0001 and *p* < 0.05, respectively, Fig.3A).

Spontaneous alternation performance decreased with age overall (*F*(4,177) = 2.88, *p* = 0.0241) and differed according to the genotype (*F*(2,177) = 4.57, *p* = 0.0117; Fig.3B). The reduced alternation rate of htau mice was significant at four months of age (*p* < 0.05 *vs*. C57Bl/6J) whereas mtau^−/−^ mice alternated less than C57Bl/6J mice at nine and twelve months of age (*p* < 0.05 in both cases). Furthermore, the alternation rate in mtau^−/−^ mice did not exceed chance level (50%) from the age of 6 months onward, indicating that they alternated at random rather than utilizing a spatial working memory strategy.

#### Open field

There were genotype (*F*(2,177) = 14.71, *p* < 0.0001) and age (*F*(4,177) = 7.20, *p* < 0.0001) effects on the total distance moved in the open field (Fig.3C). *Post-hoc* tests indicated that mtau^−/−^ mice were generally more active than C57Bl/6J mice. This was significant at four, six nine and twelve months of age (*p* < 0.05, *p* < 0.05, *p* < 0.0001 and *p* < 0.01, respectively, Fig.3C). Ratio of center to total distance moved in the open-field was unaltered by the experimental conditions (Fig.3D).

#### Novel object location and recognition tests

In the habituation trial of the novel object tests, statistical analysis revealed a main effect of genotype (*F*(2,159) = 4.45, *p* *=* 0.0132) and age (*F*(4,159) = 3.56, *p* *=* 0.0083) on total object exploration time (Fig.3E). Genotype differences were, however, not significant at the age level. Similarly, genotype (*F*(2,159) = 3.36, *p* *=* 0.0373) and age (*F*(4,159) = 4.58, *p* = 0.0016) affected object exploration time in the location trial (data not shown). This difference was due to twelve-month-old mtau^−/−^ mice which explored both objects more than age-matched C57Bl/6J mice (*p* < 0.05). In the discrimination trial, all genotypes spent the same amount of time exploring both objects (Fig.3G).

Between the three genotypes, there was no statistically significant difference in side preference of the objects before they were moved (Fig.3F). In the location trial, preference for a novel object location was statistically subject to the interaction of genotype and age (*F*(4,159) = 3.76, *p* = 0.0005; data not shown). While these genotype differences were statistically significant in most age-groups, there was no consistent pattern, which may have resulted from C57Bl/6J mice’s general poor performance in this task. Preference for a novel object shape was not significantly affected by age or genotype, although mtau^−/−^ mice performed worse than the other two genotypes in the discrimination trial overall (Fig.3H).

#### Contextual fear conditioning

**Fig. 4:**
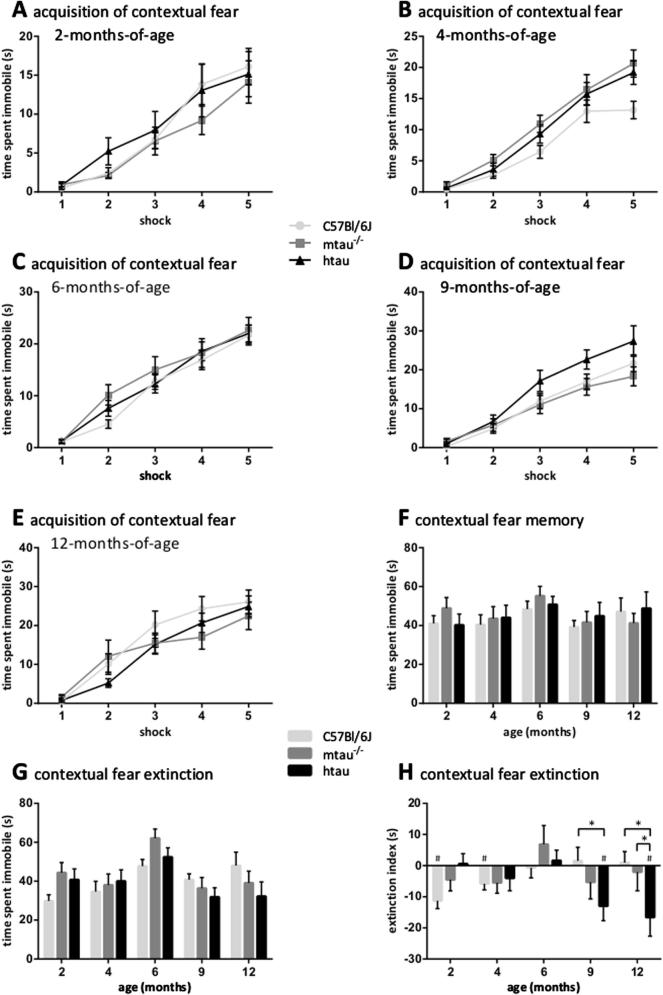
Contextual fear conditioning. Acquisition of contextual fear at (A) two, (B) four, (C) six, (D) nine and (E) twelve months of age, whereby mice received five 0.4-mA foot shocks every minute over a five-minute-long trial. The genotypes did not differ from each other in acquiring fear memory. Contextual fear memory, measured by the time the mice spent immobile in the chamber where they had received electrical foot shocks 24 (F, retention trial) or 48 (G, extinction trial) hours earlier, was unaltered according to the genotype. (G) Contextual fear extinction, expressed as a difference between immobility levels in the extinction trial relative to the retention trial was observed in two- and four-month-old C57Bl/6J and nine- and twelve-month-old htau mice as indicated by significantly negative values. KEY: ^*^p < 0.05 (planned comparisons); ^#^*p* < 0.05 compared to lack of extinction (0, one-sample *t*-test); *n* = 8–18 per age and genotype.

All experimental groups showed significantly increased immobility with repeated shock exposure indicating successful acquisition of contextual fear, which was, however, significantly altered with age (age × shock: *F*(16,676) = 3.25, *p* < 0.0001; Fig.4A–E).

Contextual fear memory, was unaltered by genotype (Fig.4F) as was immobility in the extinction trial (Fig.4G). The extinction index differed between the genotypes as a function of age (genotype × age: *F*(8,166) = 2.20, *p* = 0.0299, Fig.4H). htau mice extinguished better than C57Bl/6J mice at nine and twelve months of age (*p* < 0.05 in both cases) and better than mtau^−/−^ mice at twelve months of age (*p* < 0.05). Two- and four-month-old C57Bl/6J as well as nine- and twelve-month-old htau mice showed significant extinction (*p* < 0.05).

#### Elevated plus maze test

Performance was not significantly altered by any of the experimental conditions (data not shown).

#### Food-burrowing performance

**Fig. 5:**
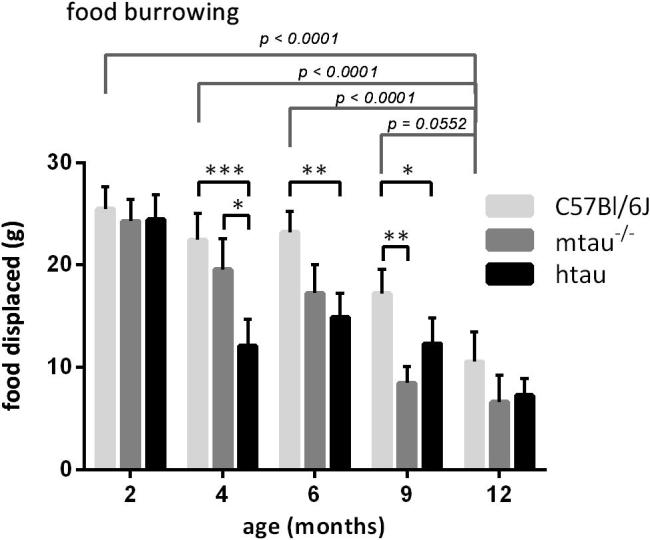
Food burrowing discriminated C57Bl/6J mice from htau mice and mtau^−/−^ mice whereby impairments caused by tau dysfunction progressed faster in htau mice. KEY: ^*^p < 0.05, ^**^p < 0.01, ^***^p < 0.0001 (planned comparisons); *n* = 8–19 per age and genotype.

Food-burrowing performance decreased significantly with age (*F*(4,187) = 23.10, *p* < 0.0001), and was altered by the genotype (*F*(2,187) = 14.59, *p* < 0.0001); whereby C57Bl/6J mice displaced more food than htau or mtau^−/−^ mice (Fig. 5). Specifically, htau mice displaced less food than C57Bl/6J mice at four , six and nine months of age (*p* < 0.0001, *p* < 0.01 and *p* < 0.05, respectively, Fig. 5). A similar trend was observed at twelve months of age (*p* = 0.0720), when performance of C57Bl/6J mice had declined significantly (*p* < 0.0001 compared to two-, four- and six-month-old C57Bl/6J mice and *p* = 0.0552 *compared to* nine-month-old mice C57Bl/6J mice. Four-month-old htau mice also burrowed less food than age-matched mtau^−/−^ mice (*p* < 0.05); while nine-month-old mtau^−/−^ mice performed more poorly than C57Bl/6J mice of the same age (*p* < 0.01).

## Discussion

The current study was conducted to further characterize the cognitive and behavioral consequences of tau dysfunction with aging in htau and mtau^−/−^ mice. As a tauopathy construct, htau mice are reported to develop pathological tau hyperphosphorylation and aggregation to NFT-like structures in neurons of the cerebral cortex and hippocampus (Andorfer et al., 2003), ultimately causing impaired tau function (Johnson and Stoothoff, 2004). But while some htau and mtau^−/−^ deficits overlap, we observed subtle differences between the two genotypes that may reflect distinct functional effects. Furthermore, while the htau phenotype appeared to be milder than the mtau^−/−^ phenotype overall, it is more severe by 6 months of age and preceded by tau hyperphosphorylation suggesting that the functional consequences of the pathological gain of tau function precede those of the loss of tau function. The delayed behavioral phenotype of mtau^−/−^ mice is in agreement with the proposal that there are compensatory mechanisms for the loss of tau in tau knockout models (e.g. increased levels of microtubule-associated protein 1A (MAP1A)) which decline with age (Ke et al., 2012).

The first major finding was that a deficit in food-burrowing performance was the most robust behavioral characteristic of htau dysfunction. The second major finding was that both htau and mtau^−/−^ mice exhibited altered performances in the spontaneous alternation task, assessing short-term working memory, and the species-specific food borrowing task; suggesting that a functional tau system is required for these paradigms. In contrast to htau deficits which were found at a four months of age, mtau^−/−^ impairments became apparent at a later stage (nine months of age) in both tasks. The two genotypes further differed from each other in that – opposed to C57Bl/6J performance – htau mice showed successful extinction of fear memory at nine and twelve months of age; while four-, nine- and twelve-month-old mtau^−/−^ mice showed impaired object recognition memory. Overall, behavioral aberrations from C57Bl/6J performances were rather subtle in htau and mtau^−/−^ mice in which specific phenotypes were confined to selected tasks and age-groups. For example, htau burrowed less food than C57Bl/6J and mtau^−/−^ mice at four months of age, entered more arms in the spontaneous alternation task at six and twelve months of age and extinguished contextual fear successfully at twelve months of age. On the other hand, mtau^−/−^ mice specifically displayed a hyperactive phenotype while weighing more than C57Bl/6J and htau mice.

The present study highlighted attenuated food burrowing as the most marked and consistent behavioral deficit of the htau behavioral phenotype compared to C57Bl/6J. Compared to mtau^−/−^ mice, the compromized burrowing performance seemed accelerated in htau mice, and was preceded by pathological tau phosphorylation. Food burrowing was the most sensitive task to identify htau mice, and the age-related difference distinguished tau overexpression from the lack of tau. The food-burrowing impairment was first noted at four months of age and was persistent at six and nine months of age. At twelve months of age the same trend was observed, but failed to reach statistical significance owing to an age-dependent performance decline of C57Bl/6J mice, which was not due to changes in locomotor activity or curiosity. This confirmed a previous observation of age-related decline in food burrowing between four- and 21-month-old C57Bl/6 mice (Hart et al., 2012). Food-burrowing behavior has been described as an ethologically oriented approach (Deacon et al., 2002) addressing an innate behavior that may relate to “activities of daily living” in humans; and may, hence, have relevance for translational research in AD (Deacon, 2009). Food-burrowing behavior has specifically been shown to respond to the integrity of both the frontal cortex and hippocampus (Deacon et al., 2002, Deacon et al., 2003) and is thus sensitive to a number of neuropathological processes. The impaired food-burrowing performance seen in both htau and mtau^−/−^ mice therefore suggests that both the loss and pathological gain of tau function affect hippocampal and fronto-cortical integrity, but the underlying mechanisms need to be elucidated. Neuroinflammation is associated with deficits in food burrowing (Teeling et al., 2010), and elevated levels of pro-inflammatory cytokines were found in the cortex of htau mice from 3 to 4 months of age (Garwood et al., 2010) which coincides with the onset of food-burrowing impairment in our study. While the slower development of food-burrowing deficit in mtau^−/−^ mice could also be related to impaired neuronal circuit formation in the cortex and hippocampus seen in tau knockouts mice and the age-related decrease in compensation of the loss of tau function by MAP1A and/or (Ke et al., 2012).

Four-month-old htau mice presented deficits in a short-term spatial memory task with lower spontaneous alternation performance than C57Bl/6J mice. Early short-term spatial memory defects of htau mice may relate to comparable observations in patients diagnosed with mild cognitive impairment which later advanced to AD (Didic et al., 2011). In the literature, however, spatial memory impairments have only been reported for twelve-month-old htau mice in the Morris water maze (Polydoro et al., 2009). The present study assessed longer-term spatial memory in the novel object location paradigm. Unfortunately, no conclusions from this trial could be drawn for htau or mtau^−/−^ mice, because C57Bl/6J mice did not discriminate between a novel or familiar object location consistently. It is currently unclear why C57Bl/6J mice did not explore the novel object location above chance. The task followed a previously established protocol (Rattray et al., 2010, Bonardi et al., 2011) with the exception of the interval between the habituation and location trial being shortened by forty minutes, theoretically improving performance in novel object recognition tasks (Sutcliffe et al., 2007).

The contextual fear-conditioning paradigm tested acquisition, retention and extinction of contextual fear. Previous reports, using a different protocol, pointed at an association between the absence of central tau and deficits in acquiring and/or retaining contextual fear memory (Ikegami et al., 2000, Ahmed et al., 2014). In the present study, mtau^−/−^ as htau mice did not display altered immobility during acquisition of fear memory – in line with one publication (Ahmed et al., 2014) – or during the retention and extinction trials. Contextual fear memory had not been tested in htau mice before this study wherein a deficit in contextual fear extinction was revealed as early as two and four months of age in both htau and mtau^−/−^ mice. This supports our previous observations that context extinction is the earliest behavioral deficit seen in mouse models of AD (Pardon et al., 2009, Rattray et al., 2009, Bonardi et al., 2011). Previously, six-month-old htau mice had shown mild impairments in a task drawing on cognitive flexibility, *i.e.* learning and re-learning of a visuospatial context (Phillips et al., 2011), consistent with the lack of extinction of contextual fear in htau mice at six months of age. But surprisingly, at nine and twelve months of age, htau mice extinguished contextual fear despite the presence of phosphorylated tau. Similar to the present study, a reduction of freezing behavior during extinction of contextual fear was seen in sixteen- but not in nine-month-old rTgTauEC mice developing NFTs composed of mutant human tau in the entorhinal cortex (Polydoro et al., 2014). This suggests that facilitation of contextual fear extinction appears with advancing age in mouse models of tau hyperphosphorylation. Given the relevance of the htau model to AD, increased forgetfulness would be a tempting interpretation for the enhanced extinction of contextual fear memory. Usually, a reduction in freezing behavior in the extinction trial is interpreted as competent re-assessing, *i.e.* a cognitively flexible approach, rather than increased forgetting of the context (Lattal and Abel, 2001, Rattray et al., 2009). However, contextual fear extinction was impaired in C57Bl/6J mice with aging, thus tau phosphorylation may have protected htau mice from this specific cognitive decline.

Arguing in favor of htau mice’s preserved memory is the concomitant performance in object discrimination which was similar to C57Bl/6J performance. Although object discrimination deficits had been reported in twelve-month-old htau mice (Polydoro et al., 2009), the present recognition memory result is consistent with one previous report using eighteen-month-old rTgTauEC mice (Chabrier et al., 2014). Differences in the protocols may have contributed to the observed discrepancies.

The present study compared htau mice to their mtau^−/−^ background mice. Since the deletion of murine tau has been shown to elicit a specific behavioral phenotype (see review by Ke et al. (2012)), this accounted for the hypothesis that the gain-of-pathological-tau function is associated with a loss-of-physiological-tau function in htau mice. Exploring the lack of functional murine tau is relevant to the research of neurodegenerative diseases such as AD, in which physiological tau becomes dysfunctional due to hyperphosphorylation (Lei et al., 2012, Morris et al., 2013). As discussed above, some of the variables that were affected by the presence of human tau, were also influenced by the lack of murine tau alone; albeit at different ages. mtau^−/−^ deficits were mainly noted at nine and twelve months of age when compensatory increases of MAP1A – present at young ages – declined at least in other mtau^−/−^ lines (Ke et al., 2012, Abbondante et al., 2014). By comparison, differences between htau and C57Bl/6J mice were often noted at four months of age when increased tau phosphorylation occurred. But whether the changes in MAP1A occurring in mtau^−/−^ mice are influenced by tau hyperphsophorylation in htau mice is unknown.

mtau^−/−^ mice were significantly heavier than C57Bl/6J and htau mice from six months of age onward. The present study’s finding of mtau^−/−^ mice’s increased body mass is in concordance with reports from twelve- to fifteen-month-old tau knock-out mice compared to their C57Bl/6J background strain (Morris et al., 2013) but in disagreement with observations of six- and twelve-month-old tau knock-out mice when compared to their C57Bl/6/SV129 hybrid controls (Lei et al., 2012). The difference in background strain may be the cause for this discrepancy, given that the 129T2 sub-strain of SV129 appears to be heavier than the C57Bl/6J strain at as early as three months of age (Andrikopoulos et al., 2005). The reason for the weight gain requires further investigations. Potential increases in MAP1A, correlated to weight gain (Takei et al., 2015), would have theoretically only occurred at a young age (Ke et al., 2012). Whether tau-insulin interactions (Doetschman, 2009, Abbondante et al., 2014) play a role in mtau^−/−^-related weight gain remains to be elucidated.

Increased body mass may affect locomotor performance (Morris et al., 2013) and was a significant covariate in the present analysis of the open field data. While the hyperactive phenotype of mtau^−/−^ mice develop with age in the open-field, together with the increased weight gain, it is unlikely the consequence of being overweight as the opposite would be expected (Morris et al., 2013). Furthermore, hyperactivity was specifically observed in the open-field. Short-lasting (five-minute-long) Y- or elevated plus maze tasks could not reveal an impact of tau absence on locomotor activity, as measured by the frequency of arm entries or the distance moved, in line with published reports (Roberson et al., 2007, Lei et al., 2012, Ahmed et al., 2014). Using arm entries in an elevated maze as a measure for locomotor activity has previously been described as relatively insensitive (Dawson and Tricklebank, 1995). Hence, arm entry measures may rather describe exploratory behavior (Tamada et al., 2010). The locomotion of tau knock-out mice in an open field over ten (Ahmed et al., 2014) or fifteen minutes (Morris et al., 2013) also did not appear to differ from C57Bl/6J’s locomotor activity. In contrast, allowing mtau^−/−^ mice to move freely through an open field for 30 min, revealed a hyperactive phenotype; agreeing with a previous report (Ikegami et al., 2000). This suggests that the hyperactive phenotype reflects a deficit in habituation to the arena.

Furthermore, mtau^−/−^ mice showed memory deficits in the spontaneous alternation test and object discrimination task at nine and twelve months of age at which htau mice were unimpaired. To the authors’ knowledge, mtau^−/−^ mice had not been tested in these paradigms before this study.

The use of non-littermate C57Bl/6J controls may have introduced a bias in genotype-related differences but is routine procedure with htau mice (Polydoro et al., 2009, Phillips et al., 2011) as they are bred on mtau^−/−^ background. The breeding strategy was based on the assumption that mtau^−/−^ mice are asymptomatic which has since then being debated (reviewed by Ke et al. (2012)). Our findings provide further evidence for a behavioral phenotype in mtau^−/−^ mice which make them unsuitable as control animals. Thus wild-type littermate would be preferable in future studies using this mouse model.

As shown above, the total absence of murine tau showed not to be essential for survival but it was not without its effects. This was evident in the mtau^−/−^ mice’s increased body mass; or in the increased incidence of systemic pathologies, such as tumours, in htau mice lacking murine tau (Levine et al., 2009). Incidentally, a third of the nine- and twelve-month-old mtau^−/−^ mice of the present study had to be excluded since they developed dermatitis leading to an excessive scratching behavior (unpublished observations). Physiological tau is also hypothesized to be necessary for neuronal maturation and maintenance: cell cultures derived from tau knock-out mice on C57Bl/6/SV129 background showed that the absence of tau was associated with abnormalities in neuronal morphology, such as dendritic length (Dawson et al., 2001). Interestingly, the authors also demonstrated that replacing murine tau with human transgenic tau returned morphometric measures, like neurite length and axonal length, to wild-type values (Dawson et al., 2001). Extrapolating these observations to the functional parameters of the present study, this may explain why htau mice presented less physiological and cognitive deviations from C57Bl/6J measurements than mtau^−/−^ mice did.

## Conclusions

The present study supports previous work indicating behavioral and cognitive impairments in htau mice when compared to C57Bl/6J controls (Polydoro et al., 2009, Phillips et al., 2011). However, it stresses on the age- and task-specific selectivity of their deficits. We clearly showed early impairments in tasks sensitive to the progression of AD-like features in htau mice, e.g. food burrowing, spontaneous alternation and extinction of contextual fear. From nine months of age onward only few aberrations from C57Bl/6J behavior were observed in htau mice; in line with reports from eight-month-old htau_SL_ mice (Chabrier et al., 2014). The behavioral phenotype of htau mice did not increase significantly in severity with age in agreement with the observation that, neither the early tau phosphorylation marker CP13, nor the pathological tau marker PHF1, showed significantly increased levels with age. This therefore suggests that the behavioral alterations observed in htau mice are a consequence of tau overpression. In favor of this argument, it was shown that increasing htau load by overexpression of mutated full-length hTau or the -4R had a limited impact of on behavior, but the effects, despite being modest, were reversed by suppressing the pathological tau species (Sydow et al., 2011, Van der Jeugd et al., 2011).

While the mtau^−/−^ phenotype was not very severe, the htau phenotype appeared even milder at later ages; and both behavioral phenotypes showed some similarities with each other: either owing to a decline in performance in C57Bl/6J mice; or owing to the absence of murine tau in the htau mice’s genotype – where the presence of human tau may compensate for the loss of murine tau to some extent; or owing to the occurrence of defunct tau systems in both mouse strains (Andorfer et al., 2003). The presence of differences in the behavioral profile of htau and mtau^−/−^ mice would however, suggest that the two genetic alterations have distinct functional effects.

Finally, our findings stress the importance of the genetic background on the phenotype of htau mice. The background-dependent phenotypic variability in genetically altered mouse models of disease is increasingly recognized. In particular, the C57BL/6j background generally yields to milder phenotypes in a number of disease models, e.g. amyotrophic lateral sclerosis (Heiman-Patterson et al., 2011), autistic spectrum disorder (Kerr et al., 2013), neurokinin-1 receptor knockout mice (McCutcheon et al., 2008), diabetes and insulin resistance (Haluzik et al., 2004), although it has occasionally been found to worsen aspects of the phenotype e.g. motor coordination in a model of Angelman syndrome (Huang et al., 2013) or amyloid plaque load in a mouse model of AD (Lehman et al., 2003). htau mice in this study were bred from the Jackson laboratory’s stock *Mapt^tm1(EGFP)Klt^* Tg(MAPT)8cPdav/J on a C57BL/6j background and their disease phenotype appears milder than previously observed in the original line bred on a heterogeneous genetic background (Andorfer et al., 2003). This suggests that the C57BL/6j background also weakens the phenotype of htau mice.

## Figures and Tables

**Table 1:** Experimental mice used in the present study. The numbers varied due to the occurrence of dermatitis in some older mtau^−/−^ mice leading to their exclusion from the study and their replacement while controlling for genotype and age factors

Mouse age	C57Bl/6J	mtau^−/−^	htau
2-months	15	14	12
4-months	15	17	15
6-months	19	13	17
9-months	15	11	12
12-months	11	8	9

**Table 2:** Results from AN(C)OVAs and mixed model analysis with repeated measures obtained from body mass measurements and behavioral and cognitive performances. KEY: df degree(s) of freedom, R residuals (single measure parametric AN(C)OVA) or denominator degrees of freedom (mixed model repeated measures analysis), F *F*-value, *p p*-value

Test	Parameter	Unit	Effect	*df*	*R*	*F*	*p*
Body mass	Mass	Grams	Age	4	188	122.90	**<0.0001**
			Genotype	2	188	21.03	**<0.0001**
			Age × genotype	8	188	2.27	**0.0245**

Spontaneous alternation	Number of arms visited	–	Body mass	1	177	4.31	**0.0393**
			Age	4	177	2.16	0.0753
			Genotype	2	177	6.93	**0.0013**
			Age × genotype	8	177	2.36	**0.0193**
							
	Alternation rate	%	Number of arms visited	1	177	2.06	0.1532
			Age	4	177	2.88	**0.0241**
			Genotype	2	177	4.57	**0.0117**
			Age × genotype	8	177	1.53	0.1498

Open field	Distance moved	Meters	Body mass	1	177	19.88	**<0.0001**
			Age	4	177	7.20	**<0.0001**
			Genotype	2	177	14.71	**<0.0001**
			Age × genotype	8	177	1.78	0.0842
	Distance moved in center	%	Body mass	1	177	1.18	0.2784
			Age	4	177	1.67	0.1580
			Genotype	2	177	1.60	0.2054
			Age × genotype	8	177	0.94	0.4854

Novel object – habituation	Exploration time	Seconds	Distance moved (open field)	1	159	0.12	0.7338
			Age	4	159	3.56	**0.0083**
			Genotype	2	159	4.45	**0.0132**
			Age × genotype	8	159	0.55	0.8158
	Preference index	-	Total exploration time (novel object habituation)	1	159	1.22	0.2713
			Age	4	159	1.70	0.1521
			Genotype	2	159	0.99	0.3748
			Age × genotype	8	159	1.38	0.2091

Novel object – location	Exploration time	Seconds	Distance moved (open field)	1	159	1.44	0.2319
			Age	4	159	4.58	**0.0016**
			Genotype	2	159	3.36	**0.0373**
			Age × genotype	8	159	0.46	0.8809
	Preference index	-	Total exploration time (novel object location)	1	159	0.13	0.7220
			Age	4	159	0.50	0.7388
			Genotype	2	159	0.44	0.6451
			Age × genotype	8	159	3.76	**0.0005**

Novel object – discrimination	Exploration time	Seconds	Distance moved (open field)	1	159	5.47	**0.0205**
			Age	4	159	3.27	**0.0131**
			Genotype	2	159	2.12	0.1234
			Age × genotype	8	159	0.41	0.9158
	Preference index	–	Total exploration time (novel object discrimination)	1	159	4.78	**0.0303**
			Age	4	159	1.44	0.2221
			Genotype	2	159	2.43	0.0917
			Age × genotype	8	159	0.77	0.6311

Contextual fear – acquisition	Time spent immobile	Seconds	Distance moved (open field)	1	168	22.94	**<0.0001**
			Age	4	168	12.17	**<0.0001**
			Genotype	2	168	2.01	0.1374
			Shock	4	676	413.44	**<0.0001**
			Age × genotype	8	168	1.37	0.2144
			Age × shock	16	676	3.25	**<0.0001**
			Genotype × shock	8	676	1.87	0.0618
			Age × genotype × shock	32	676	1.18	0.2331

Contextual fear – retention	Time spent immobile	Seconds	Total immobility (acquisition of contextual fear)	1	166	45.87	**<0.0001**
			Age	4	166	2.72	**0.0312**
			Genotype	2	166	0.35	0.7054
			Age × genotype	8	166	0.59	0.7879

Contextual fear – extinction	Time spent immobile	Seconds	Immobility during acquisition	1	166	45.87	**<0.0001**
			Age	4	166	2.72	**0.0312**
			Genotype	2	166	0.35	0.7054
			Age × genotype	8	166	0.59	0.7879
	Extinction index	Seconds	Immobility during acquisition	1	166	0.07	0.7928
			Age	4	166	2.64	**0.0357**
			Genotype	2	166	1.22	0.2983
			Age × genotype	8	166	2.20	**0.0299**

Elevated plus maze	Time spent on open arms	%	Distance moved (elevated plus maze)	1	116	5.64	**0.0192**
			Age	4	116	0.79	0.5349
			Genotype	2	116	1.54	0.2197
			Age × genotype	8	116	0.53	0.8353
	Time spent in center	Seconds	Distance moved (elevated plus maze)	1	117	67.29	**<0.0001**
			Age	4	117	1.35	0.2547
			Genotype	2	117	0.06	0.9405
			Age × genotype	8	117	0.78	0.6227

Food burrowing	Food displaced	Grams	Frequency of testing	1	187	22.68	**<0.0001**
			Age	4	187	23.10	**<0.0001**
			Genotype	2	187	14.59	**<0.0001**
			Age × genotype	8	187	1.67	0.1073

Significant main effects are highlighted in bold.

**Table 3:** Summary of key AN(C)OVA results. KEY: – no deviation from C57Bl/6J; 2 m, 4 m, 6 m, 9 m, 12 m refers to age-groups tested: two, four, six, nine, twelve months of age, respectively; ↑ increased (or improved in the case of contextual fear extinction index), ↓ decreased performance in comparison to age-matched C57Bl/6J mice

Test	Parameter	htau *vs.* C57Bl/6J		mtau^−/−^*vs.* C57Bl/6J		Fig.
Change	Age	Change	Age
Body mass	Mass	–	–	↑	6–12 m	2
		–	–	↑ *vs.* htau	9, 12 m	

Spontaneous alternation	Alternation rate *(short-term working memory)*	↓	4 m	↓	6⁎, 9, 12 m	3B

Novel object discrimination	Preference index *(recognition memory)*	–	–	↓	4, 9, 12 m⁎	3H

Contextual fear conditioning	Extinction index *(extinction of contextual fear extinction)*	↑	9, 12 m	–	–	4H
		↑ *vs.* mtau^−/−^	12 m			

Food burrowing	Food displacement *(specie-specific)*	↓	4, 6 m	↓	9 m	5
		↓ *vs.* mtau^−/−^	4 m			

⁎ Results obtained from one-sample *t-*test.
